# Utilizing EPR spectroscopy to investigate the liquid adsorption properties of bimetallic MIL-53(Al/Cr) MOF†

**DOI:** 10.1039/d3ra07952j

**Published:** 2024-01-30

**Authors:** Kavipriya Thangavel, Andrea Folli, Marcus Fischer, Martin Hartmann, Damien M. Murphy, Andreas Pöppl

**Affiliations:** a Felix Bloch Institute for Solid State Physics, Leipzig University Linnestraße 5 04103 Leipzig Germany poeppl@physik.uni-leipzig.de; b School of Chemistry, Cardiff University Main Building Cardiff CF10 3AT UK; c Net Zero Innovation Institute, Cardiff Catalysis Institute, School of Chemistry, Cardiff University Translational Research Hub, Maindy Road CF24 4HF Cardiff UK follia@cardiff.ac.uk; d Erlangen Center for Interface Research and Catalysis (ECRC), Friedrich-Alexander-Universität Erlangen-Nürnberg Egerlandstrasse 3 91058 Erlangen Germany; e National High Magnetic Field Laboratory Tallahassee Florida 32310 USA

## Abstract

The flexibility of the MIL-53(M) metal–organic framework (MOF) has been elucidated through various characterization methodologies, particularly in gas and liquid adsorption processes. However, to the best of our knowledge, there has been no prior electron paramagnetic resonance (EPR) characterization of liquid-phase adsorption in the MOF MIL-53(M), which offers insights into local geometric changes at the oxygen octahedron containing the metal ions of the framework. In this study, we investigate, for the first time, the pore transformations within the MIL-53(Al_0.99_Cr_0.01_) framework during liquid-phase adsorption using EPR spectroscopy. Our investigation concentrates explicitly on the adsorption of pure solvents, including water, methanol, ethanol, isopropanol, pyridine, and mixed water/methanol phases. The EPR spectroscopy on the (Al_0.99_Cr_0.01_) MOF has allowed us to witness and comprehend the transitions between the narrow pore and large pore phases by examining changes in the zero-field splitting parameters of the *S* = 3/2 Cr(iii) species. Of all the solvents examined, a robust and distinct spectral feature observed during methanol adsorption unequivocally indicates the pore opening.

## Abstract 2

The flexibility of the MIL-53(M) metal–organic framework (MOF) has been elucidated through EPR spectroscopy, in liquid adsorption processes.

## Graphical abstract

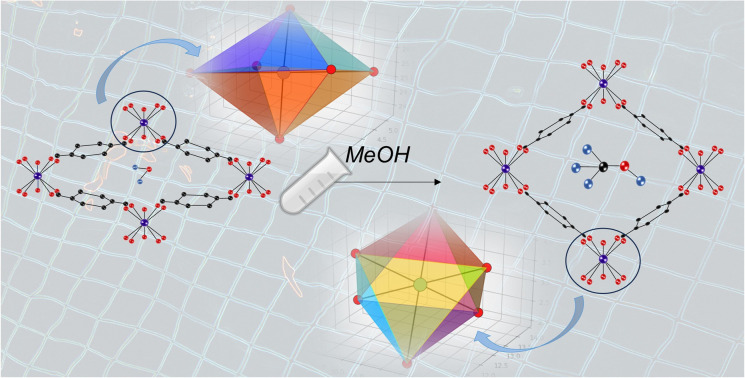

## 1 Introduction

Metal–organic frameworks (MOFs) have evoked substantial scientific attention due to their structured porous nature, which makes them exceedingly appealing for diverse applications. The exceptional properties of MOFs, including structural diversity, tunability, large internal surface area and volume, and crystal integrity, offer a promising avenue for research in various fields to address multiple challenges.^1–6^ In 2016, NuMAT Technologies commercialized MOFs as customized gas cylinders to store toxic gases, marking the first successful application of MOFs.^7^ These versatile materials exhibit immense promise in areas including but not limited to gas storage and separation, liquid purification, catalysis, sensing, dielectrics, supercapacitors, as well as energy and environmental applications.^4^ The multifaceted nature of MOFs offers a wealth of possibilities for further exploration and advancement in diverse scientific disciplines.

**Fig. 1:**
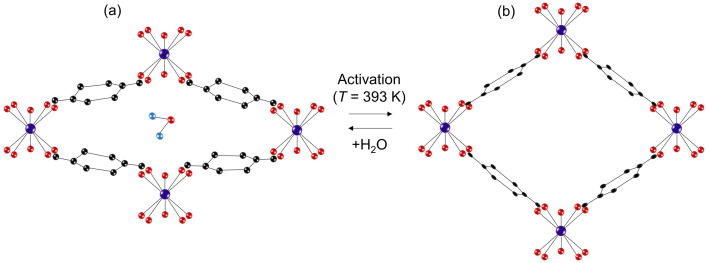
Scheme showing (a) hydrated MIL-53(M) in the np phase and (b) *T* = 393 K activated MIL-53(M) MOF in the lp phase. The scheme is modified and re-illustrated based on Bourrelly *et al.*^14^

MIL-53(M), a well-celebrated porous and flexible framework among MOFs, has gained widespread recognition for its remarkable framework pore transformations induced by gas^8^ or liquid adsorption^6^ processes and temperature variations.^9,10^ These intriguing phenomena called the breathing effect, have attracted the MOF community. MIL-53(M) is built from infinite chains of corner-sharing metal MO_4_(OH)_2_ (M = Cr(iii),^2,3^ Fe(iii),^11^ or Al(iii)^12^) octahedral units interconnected by benzene dicarboxylate (BDC) linkers resulting in a 3D MOF featuring porous channels (Fig. 1). The corner-sharing metal octahedra in the chains are linked by μ_2_-OH bridging hydroxy groups.^3^ The breathing effect in materials of the MIL-53(M) family can be controlled by the metal ion nodes^8^ and the choice of a functional group at the BDC linkers.^13^ In the case of MIL-53(Al), activation at *T* = 603 K leads to a material yielding a large pore (lp) phase with an orthorhombic crystal structure (*Imma*)^2,3,12^. Readsorption of water results in a framework with a narrow pore (np) phase having a monoclinic *C*2/*c* space group. A phase transformation into a monoclinic (*C*2/*c*) np phase can be also observed by just cooling the activated MIL-53(Al) below 150 K.^9,10^

Whereas the majority of initial studies on the framework flexibility of the MIL-53(M) family focus on gas phase adsorption applications,^8^ liquid adsorption processes have found more interest recently because of the potential of these MOF materials for separation processes.^15^ Notable examples include the separation of xylene, dichlorobenzene, chlorotoluene and nitrophenol isomers,^16–19^ as well as a variety of other aromatic compounds.^20^ Thus far, the flexibility of the MIL-53(M) MOF has been reported in the context of gas and liquid phase adsorption processes.^17,18^ Most experiments for liquid phase adsorption of MIL-53(M) materials rely on high-performance liquid chromatography (HPLC), including pulse chromatography.^16–20^ Investigations of structural changes of MIL-53(M) upon liquid phase adsorption are restricted to X-ray powder diffraction (XRD) studies of subsequently dried samples.^21^ Structural analyses in the presence of the liquid phase are rarely conducted. However, magnetic resonance spectroscopies such as nuclear magnetic resonance (NMR) and electron paramagnetic resonance (EPR) are also applicable in the liquid state and may provide structural information about the MOF framework on a local scale in the proximity of the magnetic probe as already demonstrated in NMR studies of liquid state adsorption over DUT-8 and UiO67-AcOH frameworks.^5,22^

Herein, we explore structural changes of the MIL-53(M) framework in the case of MIL-53(Al_0.99_Cr_0.01_) during liquid state adsorption for the first time by EPR spectroscopy. We focused on the adsorption of the pure solvents water, methanol, ethanol, isopropanol, pyridine, and mixed water/methanol phases. Adsorption of H_2_O and methanol was likewise studied for comparison. Here we are utilizing Cr(iii) ions with an electron spin *S* = 3/2 as a magnetic probe that substitutes isovalent framework aluminum ions in the AlO_4_(OH)_2_ octahedra.^10^ The zero-field splitting (ZFS) of the Cr(iii) ions provides information about the local symmetry of the CrO_4_(OH)_2_ octahedra and, in that way, the transformation between the np and lp of the MIL-53(Al_0.99_Cr_0.01_) framework can be examined on a local scale. This approach has been successfully demonstrated for np ↔ lp transformations triggered by temperature variations^10^ and CO_2_ gas phase adsorption.^23^ In this work, it will be extended towards the study of liquid-state adsorption processes over MIL-53(Al_0.99_Cr_0.01_).

## 2 Experimental section

MIL-53(Al_0.99_Cr_0.01_) MOF was synthesized by the hydrothermal method mentioned in the literature by Mendt *et al.*:^10^ The synthesis of (Al_0.99_Cr_0.01_) MOF was carried out using a 27 mL Teflon-lined steel autoclave. A solution composed of AlCl_3_·6H_2_O (1.30 g, 5.4 mmol), Cr(NO_3_)_3_·9H_2_O (0.0139 g, 0.034 mmol), and terephthalic acid (0.5 g, 3.0 mmol) dissolved in 5 mL of water (313 mmol) was heated to 210 °C for 48 hours. Post-filtration, a microcrystalline product displaying a subtle purple hue was obtained. The reason for substituting 1% of Cr(iii) ions is to avoid magnetic dipole–dipole and exchange interactions among EPR-active Cr(iii) species, and hence this substitution ensures the magnetic isolation of Cr(iii) species.^10^ Continuous-wave (CW) X-band (∼9.4 GHz) EPR experiments were performed on the MIL-53(Al_0.99_Cr_0.01_) utilizing Bruker EMX spectrometer fitted with Oxford instruments ESR900 cryostat. In every X-band experiment, the microwave (MW) power was kept as 2 mW (20 mW for temperature-dependent experiments), while maintaining a consistent modulation frequency of 100 kHz and a modulation amplitude of 10 G to acquire spectra without any line shape distortion and saturation. CW Q-band (∼34 GHz) EPR experiments were carried out using the Bruker EMX 10–40 spectrometer and throughout all trials, the MW power was adjusted to 1.8 mW (18 mW for temperature-dependent experiments), while maintaining a modulation frequency of 100 kHz and a modulation amplitude of 20 G.

The MOF exists in an initial hydrated state due to the adsorption of water molecules from the surrounding atmospheric conditions. The MOF underwent activation at a temperature of 393 K for 72 hours, facilitating the elimination of water molecules coordinated within the framework. To achieve water vapor adsorption, the activated sample was enclosed in an EPR tube within a sealed vessel containing boiling water for 6 h, ensuring no direct contact with the liquid phase, and enabling the sample to selectively adsorb vapor from the boiling water. Liquid adsorption studies involving methanol (MeOH), ethanol (EtOH), isopropanol (PrOH), and pyridine (py) were conducted on the hydrated MOFs. The hydrated MIL-53(Al_0.99_Cr_0.01_) sample (∼4 mg of sample in a Q-band tube of ∼1.1 mm inner diameter) was subjected to immersion within the respective liquids (10 μL) for adsorption. For the H_2_O:MeOH concentration-dependent assessment in the case of water/methanol liquid mixture adsorption, an initial addition of 10 μL of water preceded the immersion of the MIL-53(Al_0.99_Cr_0.01_) MOF (∼4 mg). Subsequently, methanol was incrementally introduced, reaching a cumulative volume of 20 μL. Q-band measurements of the obtained MOF/solvent suspensions were performed at *T* = 150 K with frozen samples as dielectric losses due to the polar solvent prevented experiments at room temperature. Otherwise, X-band experiments were feasible and done at room temperature using Q-band tubes.

Given the diverse analytical conditions applied to the material, distinct labels were employed, denoted as *H*_*x*_^*y*^ and *A*_*x*_^*y*^. Here, *H* and *A* stand for hydrated and activated states, respectively and superscript “*y*” represents either “*l*” for the lp phase or “*n*” for the np phase or “*u*” for the unidentified phase. Additionally, subscript “*x*” signifies the nature of the substance: “MeOH” for methanol, “PrOH” for isopropanol, “EtOH” for ethanol, “py” for pyridine, “H_2_O” for water, “373 K” for keeping the sample under boiling water, and “50 K” for cooled to 50 K.

Spectral simulations of the Cr(iii) EPR spectra were performed employing the EasySpin software package^24^ version 6.0.0-dev.41 installed in MATLAB R2019b, which is constructed based on the following spin Hamiltonian.
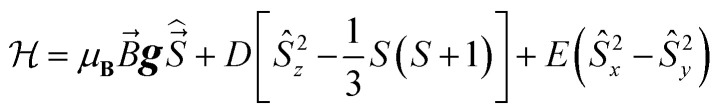
Here, in the first term, 
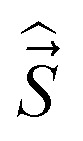
 is a matrix-valued vector operator describing the *S* = 3/2 electron spin of the Cr(iii) ion, μ_*B*_ is the electron Bohr magneton, 
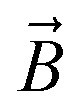
 is the applied external magnetic field vector, ***g*** is the *g*-tensor with principal values *g*_*x*_, *g*_*y*_ and *g*_*z*_, describing the electron Zeeman interaction. In the second term, 
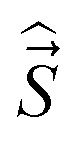
 is a matrix-valued vector operator with components *S*_*x*_, *S*_*y*_ and *S*_*z*_, describing the *S* = 3/2 spin of the Cr(iii) ion with the axial (*D*) and orthorhombic (*E*) ZFS parameters.

In order to complement the insights obtained from the EPR results, powder X-ray diffraction (PXRD) analyses were conducted for MeOH and H_2_O adsorbed samples using Panalytical X'pert diffractometer equipped with a copper anode using Cu Kα radiation (*λ* = 1.5406 Å) operating at 40 kV and 40 mA). The PXRD experiments were performed on samples subsequently dried after the adsorption process. Furthermore, the OctaDist^25^ software was employed to extract the geometric distortions evident within the Al(iii) coordination sphere, utilizing atomic coordinates sourced from the study conducted by Liu *et al.*^9^

## 3 Results and discussion

Within the MIL-53(Al) MOF, a controlled substitution of 1% Cr(iii) ion takes place at the Al(iii) ion sites. In this context, Cr(iii) serves as the designated EPR probe, facilitating the exploration of the MOF material during phase transitions induced by temperature variation or adsorption of various liquids within the pore phase.

**Fig. 2:**
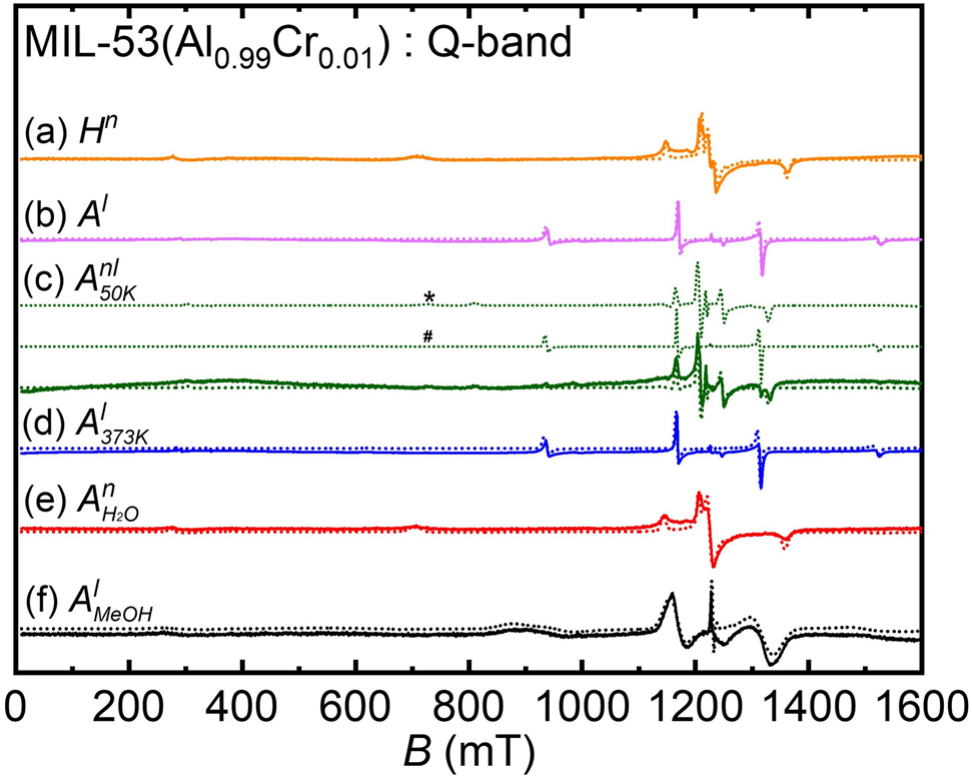
Q-band EPR data of (a) hydrated MIL-53(Al_0.09_Cr_0.01_) in np phase (*H*^n^), (b) activated MIL-53(Al_0.09_Cr_0.01_) in lp phase (*A*^l^), (c) activated sample cooled at 50 K and in a temperature triggered np/lp mixed-phase (*A*^nl^_50 K_), (d) lp phase restored by keeping the sample in the boiling water (373 K) for 15 min (*A*^l^_373 K_), (e) water vapor adsorbed for 6 h in np phase (
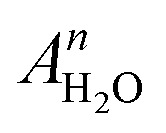
), all measured at 300 K, and (f) liquid state MeOH adsorbed sample (*A*^l^_MeOH_) measured at 150 K. (superscript n stands for np, l stands for lp, and nl stands for mixed np & lp) (solid line – experimental data, dotted line – simulation, * – 83% np phase, # – 17% lp phase).

**Fig. 3:**
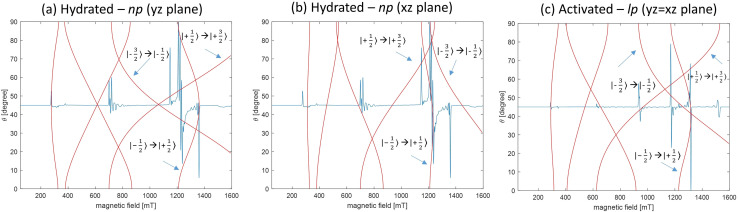
Q-band (34 GHz) EPR transition energies calculated using EasySpin for the Cr(iii) spin 3/2 quartet with (a) and (b) *g*_iso_ = 1.9775, *D* = 7.5 GHz and *E*/*D* = 0.313 corresponds to the np phase of the hydrated MIL-53(Al_0.99_Cr_0.01_) and (c) *g*_iso_ = 1.9780, *D* = 8.33 GHz and *E*/*D* = 0.005 corresponds to the lp phase of the activated MIL-53(Al_0.99_Cr_0.01_) as a function of the angle *θ* between the external magnetic field and the ZFS tensor. The unlabeled patterns correspond to the transitions Δ*m*_S_ > ±1.^27^

Prior to initiating the liquid adsorption investigations on MIL-53(Al_0.99_Cr_0.01_) MOF, we undertook a series of assessments to ascertain the inherent flexibility of this framework. The Q-band spectra recorded under various sample preparations and conditions are illustrated in Fig. 2. In this case, for the ZFS of Cr(iii) *D* ≪ *ʋ*_mw_ (microwave frequency) and, therefore, all spectra measured in the Q-band EPR experiments show three transitions originating from the spin quartets (2*S* + 1 = 4; *S* = 3/2) with a dominating central transition (*M*_*s*_ = −1/2 ↔ + 1/2; *M*_s_ – magnetic spin quantum number), split due the ZFS by second order effects, and two outer transitions (*M*_s_ = ±3/2 ↔ ± 1/2). Within the framework of MIL-53(Al_0.99_Cr_0.01_), the ZFS phenomenon emerges as a pivotal tool for comprehending the intricacies of phase transformations. This sensitivity arises from the responsiveness of ZFS to even small variations of the crystal field, which stems from the distortion of the oxygen octahedra in whose center the Cr(iii) ions are situated.^10,23^ Specifically, the axial ZFS parameter, denoted as *D*, quantifies the tetragonal distortion observed in the octahedral configuration. On the other hand, the rhombic ZFS parameter, labeled as *E* (See ESI, Fig. S3†), exhibits high sensitivity to orthorhombic distortions or even lower symmetry deviations present within the local crystal field environment.^10^ In the presence of water molecules, CrO_4_(OH) octahedral units undergo distortion with np phase, attaining increased symmetry with lp phase subsequent to water removal *via* an activation process, resulting in a reduction of the M–O bond distance (See ESI, Fig. S2 and Table S1†). The distortion parameters of AlO_4_(OH)_2_ obtained *via* OctaDist software^25^ for comparison are given in Table 1. The angular-dependent traces shown in Fig. 3 obtained for the hydrated large pore and activated narrow pore distinctly illustrate the impact of the *D* and *E* parameters (Table 2) on the spectral feature in the Q-band Cr(iii) EPR powder spectra.

**Table 1:** Distortion parameters of AlO_4_(OH)_2_ octahedral unit extracted utilizing OctaDist software. Where *D*_mean_ – average metal–ligand distances in the octahedral coordination sphere, *ζ* – the average of the sum of the deviation of 6 unique metal–ligand bond lengths around the central metal atom from the average value (*D*_mean_), *Σ* – the sum of the deviation of 12 unique *cis* ligand–metal–ligand angles (*ϕ*_i_) from 90°, *Θ* – the sum of the deviation of 24 unique torsional angles between the ligand atoms on opposite triangular faces of the octahedron viewed along the pseudo-threefold axis (*θ*_i_) from 60°.^25^

Pore phase	Corresponding state	*D*_mean_ (Å)	*ζ* (Å)	*Σ* (°)	*Θ* (°)
np	*H* ^n^	1.9228	0.3318	42.6535	130.7509
lp	*A* ^l^	1.8813	0.2999	31.5347	85.9756

**Table 2:** The spin Hamiltonian parameters *g*_iso_, *D*, Δ*D*, *E/D* of Cr(iii) ions in MIL-53(Al_0.99_Cr_0.01_) at different temperatures and vapour/liquid adsorption treatments (a – hydrated, b – activated, c–cooled at 50 K, d – heated by boiling water, e – water vapour adsorbed, f – subsequently MeOH adsorbed)

	Species	Pore phase		*T* (K)	*g*_iso_	*D* (GHz)	Δ*D* (GHz)	Δ*E* (GHz)	*E/D*
(a)	*H* ^n^	np	300		1.9775(3)	7.50(10)	0.40(5)	0.19(4)	0.313(2)
(b)	*A* ^l^	lp	300		1.9780(5)	8.33(6)	0.30(5)	<0.01(1)	< 0.005(5)
(c)	*A* ^nl^_50 K_	lp (17%)	300		1.9770(5)	8.35(5)	0.20(4)	<0.01(1)	< 0.005(5)
		np (83%)			1.9770(5)	7.00(9)	0.35(3)	0.10(5)	0.229(5)
(d)	*A* ^l^_boil_	lp	300		1.9780(5)	8.33(5)	0.30(6)	<0.01(1)	< 0.005(5)
(e)	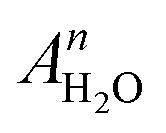	np	300		1.9775(5)	7.45(10)	0.40(2)	0.10(5)	0.330(5)
(f)	*A* ^l^_MeOH_	lp	150		1.9775(5)	9.00(12)	1.80(2)	0.40(6)	0.044(2)

The ZFS parameters *D* and *E* and the strain parameters Δ*D* and Δ*E* were determined by spectral simulations of the experimental spectra. The obtained spin Hamiltonian parameters corresponding to distinct phases (lp and np) of the various samples are presented in Table 2. In general, Cr(iii) species with almost axially symmetric ZFS tensors and parameters *D* > 8 GHz, *E/D* < 0.05 are indicative for the lp phase, whereas rhombic ZFS tensors with *D* < 7.1 GHz, *E/D* > 0.18 have been measured for the np phase during CO_2_ gas phase adsorption experiments and in activated samples at low temperatures.^10,23,26^

In Fig. 2a, the EPR spectrum of a hydrated sample (*H*^n^) is presented, with Cr(iii) ZFS parameters indicating its np phase. Subsequently, activation of the material at a temperature of 393 K in the vacuum induced the removal of water molecules embedded within the framework (*A*^l^). This thermal treatment prompted a transition of the MOF from the np phase to the lp phase, as demonstrated in Fig. 2b.^10^ Further, the EPR tube housing the sample was subjected to a temperature of 50 K for a duration of one hour, and subsequent measurements were conducted at room temperature (RT). Notably, this manipulation led to a significant phase transition, with 83% of the material transforming from the lp phase to the np phase, as depicted in Fig. 2c. To reinstate the lp phase, the EPR tube containing the material was heated at 373 K in boiling water for a duration of 15 minutes (Fig. 2d). Subsequent to this treatment, the material absorbed water vapor and reverted fully to its initial np phase (Fig. 2e). Further, the activated material was suspended within liquid MeOH and subjected later to measurements at a temperature of 150 K. Intriguingly, it is noteworthy that the material experienced a transformation back into the lp form, albeit with more pronounced *D* strain effects, even in the presence of water molecules in the suspension (Fig. 2f). The ZFS splitting parameters including strain parameters of Cr(iii) ions were estimated from spectral simulations of the Q-band spectra in Fig. 2 and summarized in Table 2. The phase transformation from the np to the lp phase upon suspending the hydrated MIL-53(Al_0.99_Cr_0.01_) MOF in MeOH was verified by PXRD experiments (See ESI, Fig. S1†).

**Fig. 4:**
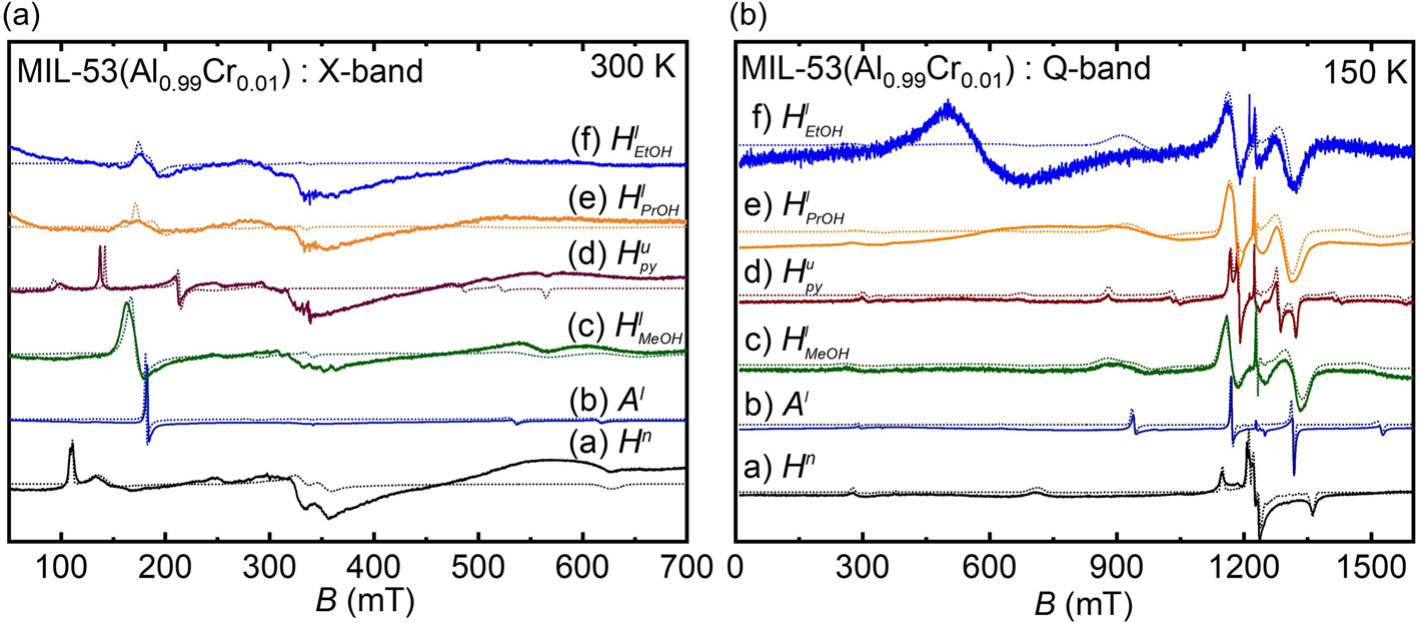
(a) X-band and (b) Q-band EPR studies at different liquid adsorptions/states (*H*^l^_EtOH_ – Ethanol, *H*^u^_PrOH_– isopropanol, *H*^u^_py_–pyridine, *H*^l^_MeOH_ – methanol, *A*^l^ – activated, *H*^n^–hydrated, solid line – experimental data, dotted line – simulation).

The successful verification of the np → lp phase transformation through liquid-state MeOH adsorption motivated us to extend our investigation to other liquids. Consequently, we conducted additional liquid state adsorption experiments on hydrated MIL-53(Al_0.99_Cr_0.01_) MOF samples using MeOH, py, PrOH, and EtOH as test liquids and employing X- and Q-band EPR spectroscopy. The resulting X- and Q-band EPR spectra are exhibited in Fig. 4. The spectra obtained for the activated as well as the hydrated samples were likewise included in Fig. 4 for comparison. We like to emphasize that the Cr(iii) Q-band spectra, where *D* ≪ *ʋ*_mw_ holds, allow for a precise determination of the axial ZFS parameter *D* from the central *M*_s_ = −1/2 ↔ + 1/2 transition and if resolved the outer *M*_s_ = ±3/2 ↔ ± 1/2 transitions. Otherwise, X-band experiments (*D* >> *ʋ*_mw_) are particularly sensitive to variations in the *E/D* ratio.

A single low field signal at about 170 mT is typical for *E/D ∼*0 of the almost axially symmetric Cr(iii) species in the lp phase whereas the splitting of the 170 mT signal and appearance of additional signals at lower field (110–130 mT) indicate ratios of *E/D* > 0.15 and the presence of the np phase (Fig. 4a). EtOH and PrOH liquid state adsorption results in strong line-broadening effects of the EPR spectra due to *D* strain up to Δ*D* = 2 GHz and consequently lower signal-to-noise ratios. However, the estimated parameters *D* and *E/D* (Table 2) suggest a transition from the np to a lp phase upon suspension of the hydrated MOF in the two alcohols. We denote these lp phases as *H*^l^_EtOH_ and *H*^l^_PrOH corresponding to EtOH and PrOH adsorptions, respectively_. We have to note that the Q-band spectrum of the EtOH adsorbed sample recorded at 150 K exhibits an additional broad signal at 550 mT of unknown origin. Similar signals were observed for the PrOH-adsorbed at somewhat lower temperatures (See ESI, Fig. S5c†). These signals might indicate the formation of different sizes of antiferromagnetically coupled Cr(iii) species and consequently the destruction of some MOF crystals in the alcohol suspensions. A minor hump between ∼200 mT to ∼550 mT is evident even in the activated np phase sample cooled at 50 K and subsequently measured at RT (*A*^nl^_50 K_ in Fig. 2a). Additionally, this hump diminishes during measurement at 373 K, a pattern anticipated to persist upon the adsorption of EtOH and PrOH, and is expected to dissipate with a rise in temperature.

In contrast, py adsorption leads to Cr(iii) species with distinctly lower *D* values and larger *E/D* ratios (Table 3). But both are not within the parameter ranges which are known for the ZFS parameters of chromium ions in either the lp or np phase of MIL-53(Al_0.99_Cr_0.01_).^10,23^ Thus, we designate it as an unidentified phase (*H*^u^_py_). The *D* strain for the *H*^u^_py_ phase is low, thus it resembles a highly ordered phase comparable to the lp phase of the activated material.

**Table 3:** The spin Hamiltonian parameters *g*_iso_, *D*, Δ*D*, *E/D* of *S* = 3/2 Cr(iii) ion MIL-53(Al_0.99_Cr_0.01_) at different liquid adsorption treatments for the X- and Q-band EPR techniques (measured at *T* = 300 K for X band and *T* = 150 K for Q-band) in comparison with hydrated and activated phases

Species	Liquids/state	*g*_iso_	*D* (GHz)	Δ*D* (GHz)	Δ*E* (GHz)	*E/D*
*H* ^l^_EtOH_	EtOH	1.975(3)	8.50(5)	2.01(4)	0.05(5)	0.02(4)
*H* ^l^_PrOH_	PrOH	1.978(5)	8.00(2)	2.01(4)	0.10(2)	0.04(1)
*H* ^u^_Py_	py	1.978(5)	7.66(6)	0.11(2)	0.03(2)	0.11(5)
*H* ^l^_MeOH_	MeOH	1.978(5)	8.45(12)	1.10(2)	0.40(1)	0.01(1)
*A* ^l^	Activated	1.9780(5)	8.33(5)	0.30(5)	<0.01(1)	< 0.005(5)
*H* ^n^	Hydrated	1.9775(5)	7.50(10)	0.40(5)	0.19(4)	0.313(2)

**Fig. 5:**
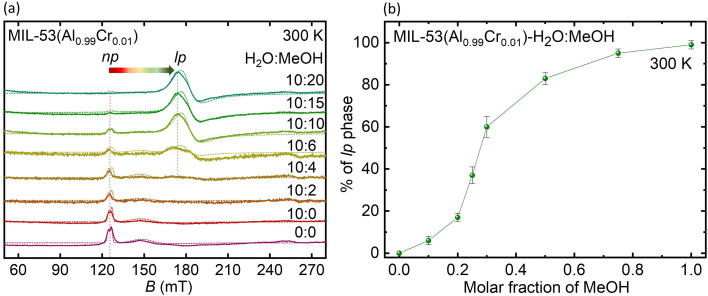
(a) X-band EPR spectra of MIL-53(Al_0.99_Cr_0.01_) recorded at *T* = 300 K for liquid state adsorption of MeOH in an H_2_O/MOF suspension (the numerical values indicate the ratio of H_2_O/MeOH in μL, dotted line – simulation) and (b) isotherm of MeOH adsorption as derived from the intensity of the Cr(iii) signals of the lp phase.

Because MeOH liquid state adsorption resulted in samples with the best-resolved spectra and a clearly assignable lp phase, we continued by examining how its adsorption varies with different concentrations in the presence of water. These experiments were performed at X-band on the hydrated MIL-53(Al_0.99_Cr_0.01_) samples at room temperature. Initially, the hydrated MIL-53(Al_0.99_Cr_0.01_) is in its np phase due to the interaction between water and the host framework. Sequential liquid state MeOH adsorption transforms the MOF into its lp phase as indicated by the change in the low field Cr(iii) signals (Fig. 5a). The np → lp phase transformation is evidenced by the disappearance of the signal at ∼130 mT of the Cr(iii) ions in the np phase and the accompanied emergence of the 170 mT signal of the lp phase with increasing MeOH content in the suspension. Upon close examination of Fig. 5a, it becomes evident that introducing only 2 μL (20%) of MeOH into the water solution results in a broadening of the signal of the np phase at ∼130 mT and the onset of the lp phase signal at ∼170 mT as indicated by a subtle change of the baseline in this spectral region. These spectral changes point to the emergence of the lp phase (∼6%) at low MeOH concentrations in the H_2_O:MeOH mixture. However, the pivotal transition happens with the subsequent addition of MeOH, with complete pore transformation achieved at the 20 μL of MeOH adsorption.

It is worth highlighting that water either retains the np phase or converts it from the lp to the np phase. On the other hand, the addition of MeOH causes the np → lp phase transformation, even when water is present, a phenomenon recognized as selective liquid adsorption. Assuming that the intensity ratio of the Cr(iii) subspectra corresponding to the lp and np phases as determined by spectral simulations (Table S2†) of the X-band spectra in Fig. 5a resemble the ratio of the volume fractions of the two phases an adsorption isotherm can be derived from the EPR results. Fig. 5b illustrates such an isotherm for MeOH liquid state adsorption depicting the percentage of the lp phase upon introducing MeOH stepwise to the suspended MOF material while 10 μL of H_2_O is concurrently present.

Bourrelly *et al.*^14^ proposed a dual classification of interactions between water molecules and host species. These interactions can be categorized as follows: interaction (1) involves hydrogen bonding between the protons of the μ_2_-OH group and the oxygens O_w_^−^ of the water molecules. On the other hand, interaction (2) encompasses hydrogen bonding between the protons of water molecules and the oxygen atoms of the carboxylate linker, which are situated in the upper and lower chains of the MIL-53 structure. And interaction (3) comprises the strong hydrogen bonds between the adsorbed water molecules. The later hydrogen bond network along the one-dimensional channels of the MIL-53 framework leads to an ordered arrangement of the water molecules and this guest–guest interaction stabilizes the np phase of the MOF.^14^ In the case of the guest molecule being MeOH, instead of interaction (3) an additional interaction (4) becomes relevant. This interaction entails a van der Waals C–C interaction occurring between the alkyl groups of the alcohol molecule and the aromatic rings present within the framework. This interaction is established between methanol and the carboxylic group within the framework structure. Simultaneously, within interaction 2, a noteworthy distinction arises in the case of MeOH adsorption comparison to the water/host interaction. Specifically, the hydrogen bonding involving the proton of methanol and the oxygen atoms of the carboxylate linker is observed exclusively within the upper or the lower chain of the structure. Conversely, when considering water molecules as guests, this hydrogen bonding is established within both the upper and lower chains of the framework. Additionally, it is noteworthy that MIL-53 preferentially adsorbs alcohol over H_2_O in liquid-phase adsorption. Presumably, this is caused by the higher adsorption enthalpies of MeOH (−60 kJ mol^−1^) and EtOH (−65 kJ mol^−1^) *versus* H_2_O (−57 kJ mol^−1^).^14^

Furthermore, Bourrelly *et al.*^14^ documented a discernible phenomenon of selective EtOH adsorption over water within the context of hydrated MIL-53(Cr), particularly evident when employing a vapour phase mixture of EtOH and water. The low-temperature EPR experiments (depicted in Fig. 4b, S4d and S5d†) confirm that EtOH adsorption results in pore expansion, accompanied by a notably large (>8.00 GHz) ZFS value (Table 2).

In the case of pyridine adsorption on MIL-53(Fe), Millange *et al.*^21^ reported that the unit cell experiences partial expansion relative to the hydrated phase, forming hydrogen bonds between N donors and OH framework atom.^21^ Our EPR observations on pyridine adsorption evidence the coordination with the framework, inferred from the change in the spectral feature, while the value of ZFS does not indicate the formation of the lp phase in the presence of py. The ZFS parameters for pyridine adsorbed samples are intermediate to the typical parameter range of the np and lp phase and might be attributed to a highly ordered framework with a partially opened pore system.

## 4 Conclusion

The application of EPR spectroscopy has proven to be a valuable tool for gaining insights into the pore phase transformations occurring within the MIL-53(Al_0.99_Cr_0.01_) framework during the adsorption of various pure solvents, such as water, methanol, ethanol, isopropanol, pyridine, and mixed water/methanol phases. This local method has enabled us to observe and understand the np ↔ lp phase transformations by monitoring the alterations in the ZFS parameters of the *S* = 3/2 Cr(iii) species. Concentration-dependent water/methanol adsorption isotherm ensured the selective liquid adsorption property of the MOF. The successful utilization of EPR spectroscopy for the first time during solvent adsorption processes in this MOF has contributed significantly to our comprehension of the dynamic structural changes within the framework. Notably, the observed EPR spectroscopic findings are consistent with the previously reported XRD results, reinforcing the validity and coherence of EPR outcomes.

## Author contributions

Kavipriya Thangavel: EPR experiments; formal analysis; investigation; writing – original draft; software; methodology; conceptualization; Andrea Folli: formal analysis; investigation; methodology. Marcus Fischer: XRD experiments and analysis; synthesis of MOF. Martin Hartmann: methodology; resources. Damien M. Murphy: formal analysis; resources; supervision. Andreas Pöppl: methodology; formal analysis; resources; writing; conceptualization; supervision.

## Conflicts of interest

There are no conflicts to declare.

## Supplementary Material

RA-014-D3RA07952J-s001
