# Supplementary material for: Utilizing EPR spectroscopy to investigate the liquid adsorption properties of bimetallic MIL-53(Al/Cr) MOF
Source: RSC Adv. 2024 Jan 30;14(6):4244–51. doi: 10.1039/d3ra07952j (PMC10826799; doi:10.1039/d3ra07952j)
Supplement: RA-014-D3RA07952J-s001 [file RA-014-D3RA07952J-s001.pdf]

Electronic Supplementary Material (ESI) for RSC Advances.
This journal is © The Royal Society of Chemistry 2024

SUPPORTING INFORMATION

Utilizing EPR Spectroscopy to Investigate the Liquid Adsorption Properties of Bimetallic MIL-53(Al/Cr) MOF

Kavipriya Thangavel[1, 2], Andrea Folli[3]*, Marcus Fischer[4], Martin Hartmann[4], Damien M Murphy[2] and Andreas Pöppl[1]*

[1]*Felix Bloch Institute for Solid State Physics, Leipzig University, Linnestraße 5, 04103 Leipzig, Germany*

[2]*School of Chemistry, Main building, Cardiff University, Cardiff CF10 3AT, United Kingdom*

[3]*Net Zero Innovation Institute, Cardiff Catalysis Institute, School of Chemistry, Cardiff University, Translational Research Hub, Maindy Road, CF24 4HF, Cardiff, UK*

[4]*Erlangen Center for Interface Research and Catalysis (ECRC), Friedrich-Alexander-Universität Erlangen-Nürnberg, Egerlandstrasse 3, 91058, Erlangen, Germany*

Table of contents

1. **XRD data and structural information of MIL-53($Al_{0.99}Cr_{0.01}$) ……….…….2**
2. **Simulated Cr(III) EPR powder spectra with *E* dependence trend for fixed *D* value ……….…….4**
3. **Auxilliary temperature dependent EPR data of MIL-53($Al_{0.99}Cr_{0.01}$) ………5**

S1. XRD data of MeOH adsorbed MIL-53($Al_{0.99}Cr_{0.01}$)

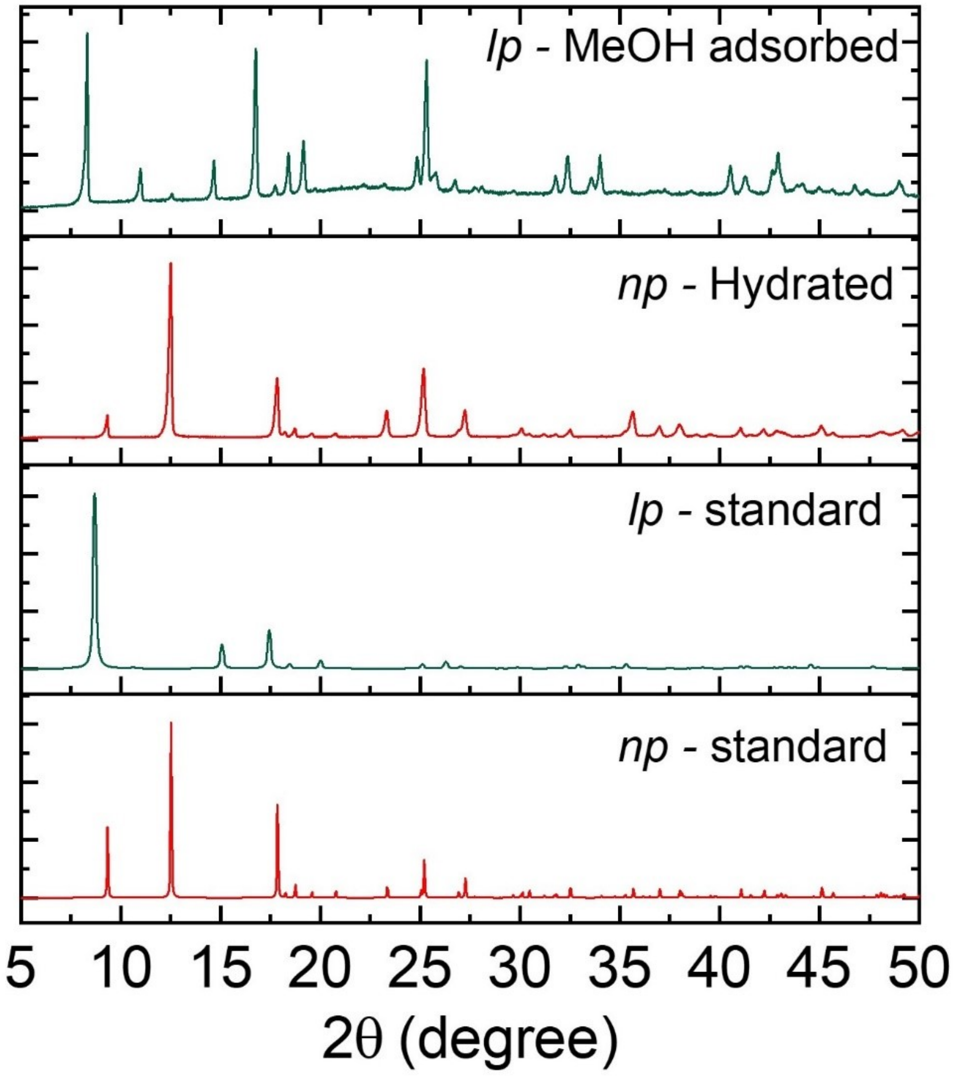

Figure S1. Theoretical PXRD patterns of the *np* and *lp* phase of MIL-53(Al) compared with the hydrated MIL-53($Al_{0.99}Cr_{0.01}$) and subsequently in MeOH adsorbed MOF. Theoretical patterns are calculated from the crystallographic data taken from Liu *et al*.[1]

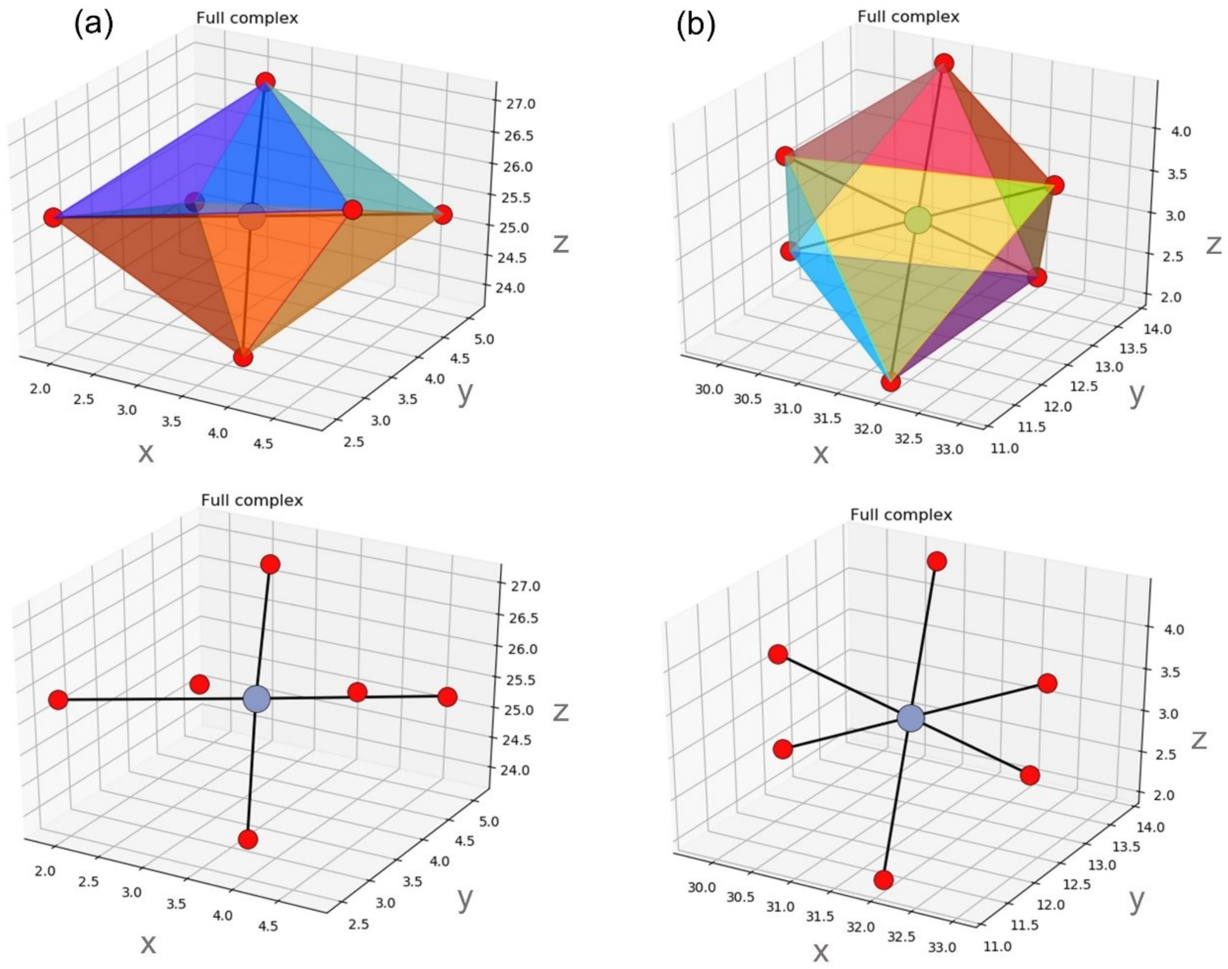

Figure S2. $AlO_4(OH)_2$ octahedral unit of (a) hydrated (*np* phase) and (b) activated (*lp* phase) of MIL-53(Al) extracted from the OctaDist software.[2]

Table S1: The distance between the metal ion and the surrounded oxygen in the $AlO_4(OH)_2$ octahedral unit for the *np* and *lp* phase of MIL-53($Al_{0.99}Cr_{0.01}$) extracted from the OctaDist software.[2]

Bond	Bond distance (Å)	
	***np* phase (hydrated)**	***lp* phase (activated)**
Al-O1	1.810742	1.806349
Al-O2	2.003136	1.918834
Al-O3	1.912103	1.918827
Al-O4	1.924856	1.918837
Al-O5	1.879603	1.806358
Al-O6	2.006225	1.918830

S2. Simulated Cr(III) EPR powder spectra with *E* dependence trend for fixed *D*-value

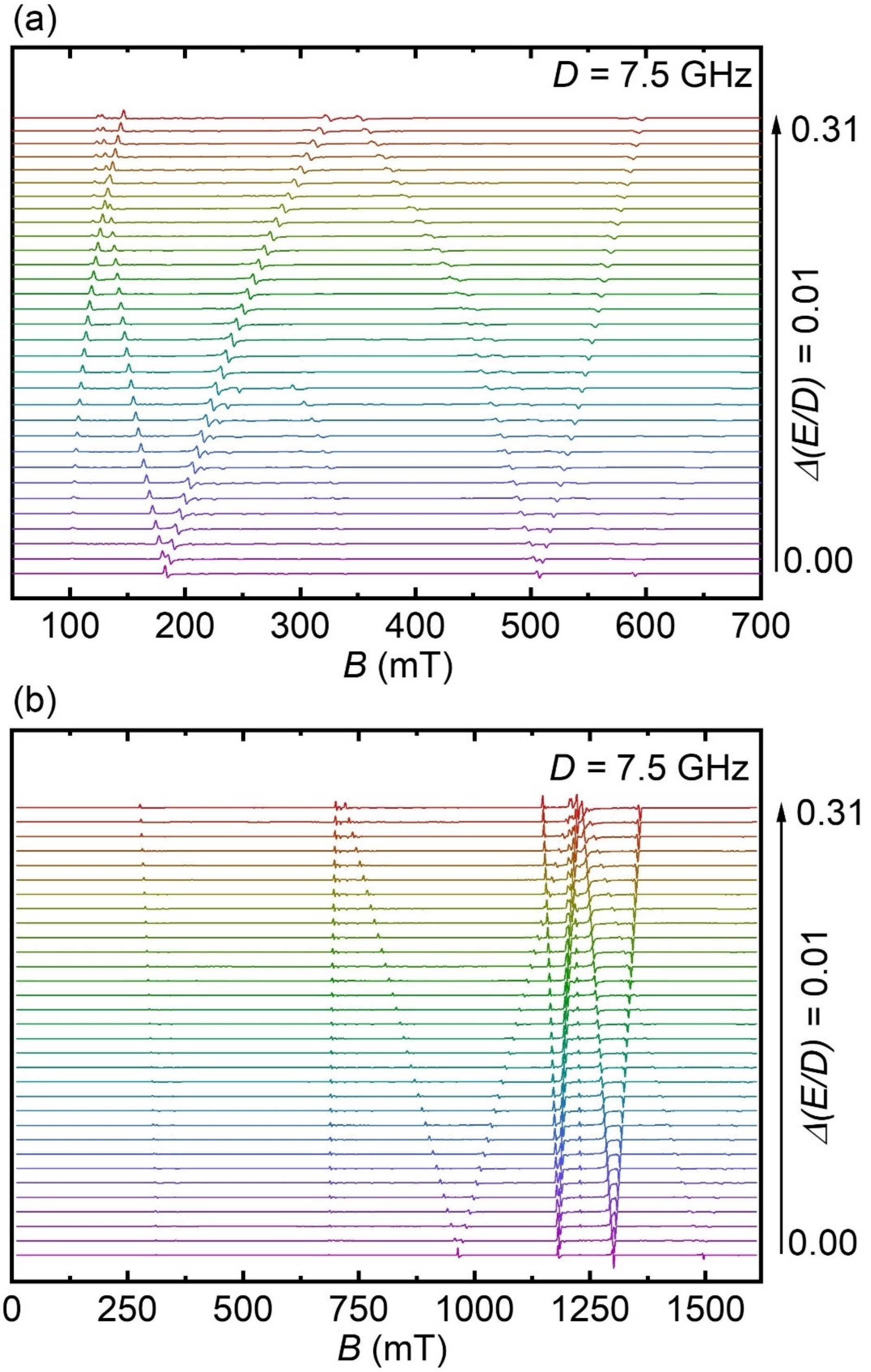

Figure S3. *E* dependence trend with *D* = 7.5 GHz for the (a) X- (9.4 GHz) and (b) Q-band (34 GHz) frequencies.

S3. Auxiliary temperature-dependent EPR data of $MIL\text{-}53(Al_{0.99}Cr_{0.01})$

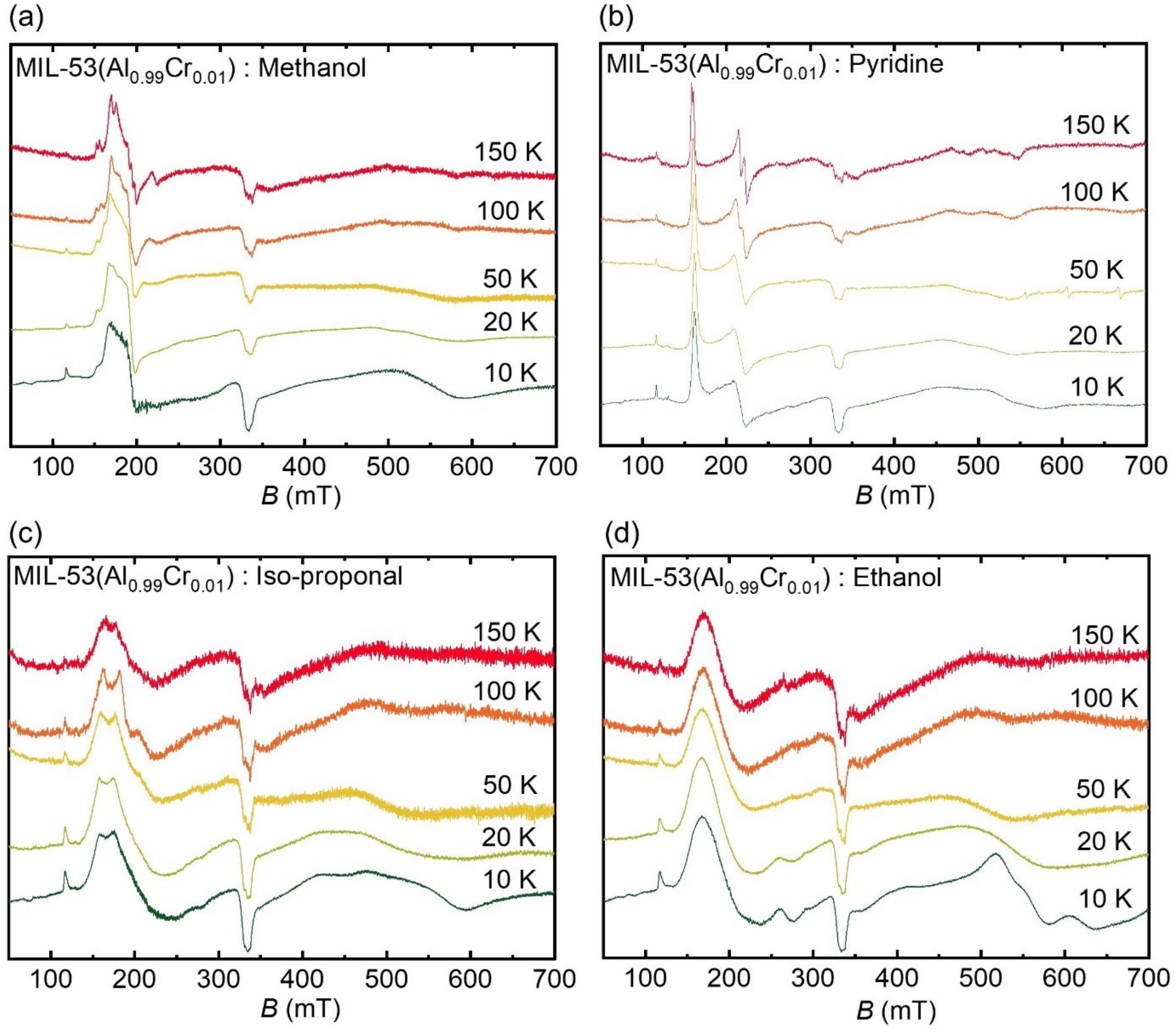

Figure S4: Temperature-dependent X-band EPR experiments of $MIL\text{-}53(Al_{0.99}Cr_{0.01})$ with (a) MeOH, (b) py, (c) PrOH and (d) EtOH liquid adsorption

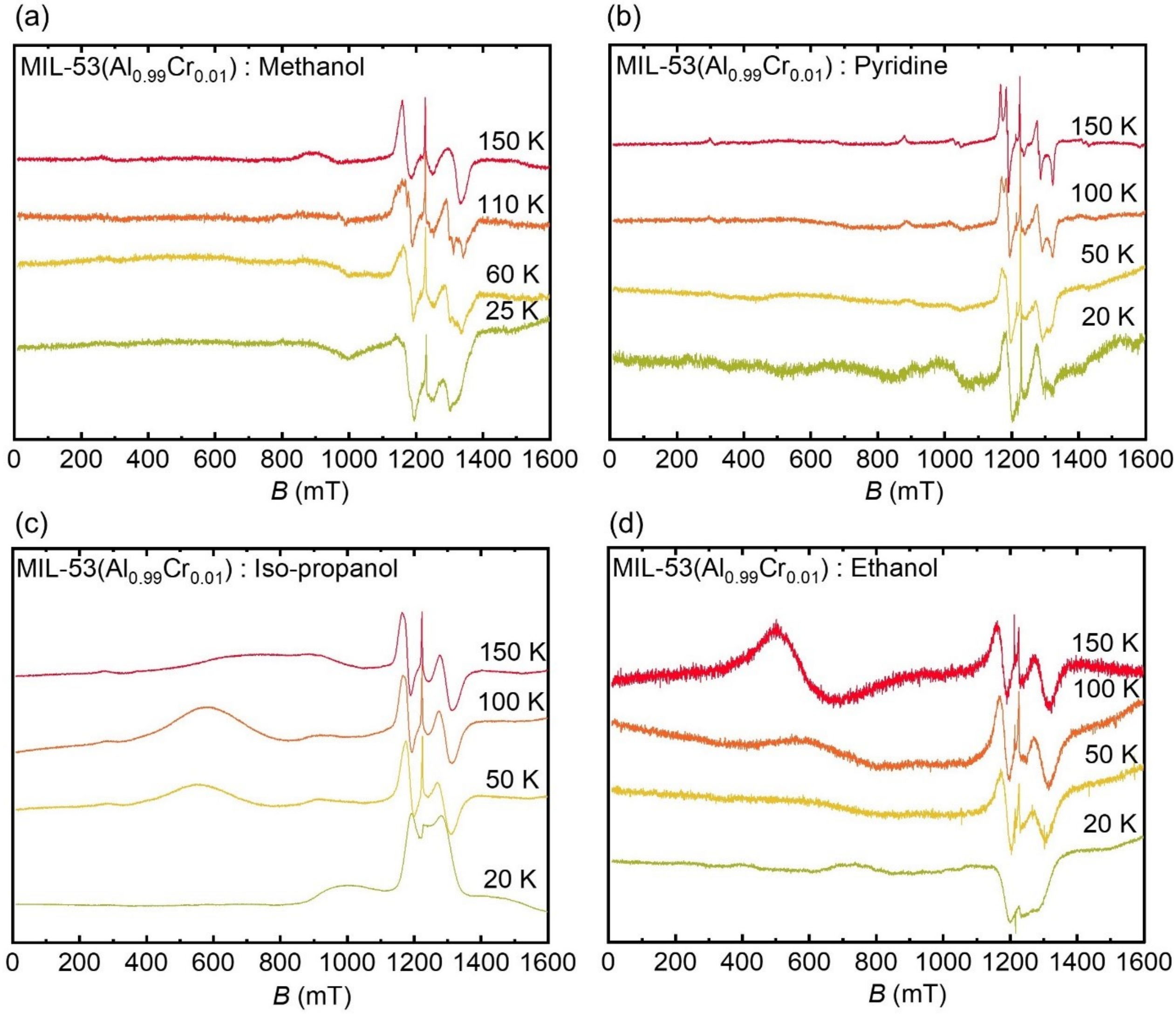

Figure S5: Temperature-dependent Q-band EPR experiments of MIL-53($Al_{0.99}Cr_{0.01}$) with (a) MeOH, (b) py, (c) PrOH and (d) EtOH liquid adsorption

Table S2: The spin Hamiltonian parameters g_{iso}, D, ΔD, ΔE, E/D of S = 3/2 Cr(III) ion in hydrated MIL-53($Al_{0.99}Cr_{0.01}$) MOF measured by X-band EPR during H_2O/MeOH adsorption at different concentrations (measured at T = 300 K)

Species (H_2O:MeOH)	g_{iso}	D (GHz)	ΔD (GHz)	ΔE (GHz)	E/D
(a) Hydrated	1.977(1)	7.50(10)	0.40(5)	0.19(4)	0.313(2)
(b) 10:0 *np* (100%)	1.975(3)	7.50(2)	0.50(5)	<0.00(1)	0.313(2)
(c) 10:2 *np* (94%)	1.975(3)	7.50(2)	0.50(5)	<0.00(1)	0.313(2)
lp (6%)	1.975(3)	7.50(2)	0.50(5)	0.30(4)	0.313(2)
(d) 10:4 *np* (83%)	1.975(3)	7.50(2)	0.40(5)	0.19(5)	0.313(3)
lp (17%)	1.978(3)	8.50(5)	1.10(5)	0.30(3)	0.017(3)
(e) 10:5 *np* (63%)	1.975 (3)	7.50(2)	0.55(2)	0.19(5)	0.313(3)
lp (37%)	1.978(3)	8.50(5)	1.10(5)	0.30(3)	0.017(3)
(f) 10:6 *np* (40%)	1.975(5)	7.40(2)	0.70(8)	0.19(5)	0.318(5)
lp (60%)	1.978(3)	8.50(5)	1.10(5)	0.30(3)	0.017(3)
(g) 10:10 *np* (17%)	1.975(3)	7.50(2)	0.40(5)	0.19(5)	0.313(3)
lp (83%)	1.978(3)	8.50(5)	1.10(5)	0.30(3)	0.017(3)
(h) 10:15 *np* (5%)	1.975(3)	7.50(2)	0.40(5)	0.19(5)	0.313(3)
lp (95%)	1.978(3)	8.50(5)	1.10(5)	0.30(3)	0.017(3)
(i) 10:20 *lp* (100%)	1.978(1)	8.45(12)	1.10(2)	0.40(5)	0.011(4)

Table S3: The information about the unit cell volume of MIL-53(M) upon adsorption for different solvents from the reported literature

Adsorbents	Phase	Volume unit cell ($Å^3$)	Metal ion	Reference
Empty (unactivated)	*np*	886	Al(III)	Liu *et al.*[1]
Empty (activated)	*lp*	1480	Cr(III)	Bourrelly *et al.*[3]
Pyridine	*lp*	1395	Al(III)	Vougo *et al.*[4]
Methanol	*lp*	1538	Cr(III)	Bourrelly *et al.*[3]
Water	*np*	1017	Cr(III)	Bourrelly *et al.*[3]

References:

1 Y. Liu, J.-H. Her, A. Dailly, A. J. Ramirez-Cuesta, D. A. Neumann and C. M. Brown, Reversible structural transition in MIL-53 with large temperature hysteresis, *Journal of the American Chemical Society*, 2008, **130**, 11813–11818.

2 R. Ketkaew, Y. Tantirungrotechai, P. Harding, G. Chastanet, P. Guionneau, M. Marchivie and D. J. Harding, OctaDist: a tool for calculating distortion parameters in spin crossover and coordination complexes, *Dalton transactions (Cambridge, England : 2003)*, 2021, **50**, 1086–1096.

3 S. Bourrelly, B. Moulin, A. Rivera, G. Maurin, S. Devautour-Vinot, C. Serre, T. Devic, P. Horcajada, A. Vimont, G. Clet, M. Daturi, J.-C. Lavalley, S. Loera-Serna, R. Denoyel, P. L. Llewellyn and G. Férey, Explanation of the adsorption of polar vapors in the highly flexible metal organic framework MIL-53(Cr), *Journal of the American Chemical Society*, 2010, **132**, 9488–9498.

4 M. Vougo-Zanda, J. Huang, E. Anokhina, X. Wang and A. J. Jacobson, Tossing and turning: guests in the flexible frameworks of metal(III) dicarboxylates, *Inorganic chemistry*, 2008, **47**, 11535–11542.
